# Perceived study-induced influence on the control group in a randomized controlled trial evaluating a complex intervention to promote psychosocial well-being after stroke: a process evaluation

**DOI:** 10.1186/s13063-021-05765-w

**Published:** 2021-11-27

**Authors:** Margrete Mangset, Gabriele Kitzmüller, Anne S. Evju, Sanne Angel, Lena Aadal, Randi Martinsen, Berit Arnesveen Bronken, Kari J. Kvigne, Line K. Bragstad, Ellen Gabrielsen Hjelle, Unni Sveen, Marit Kirkevold

**Affiliations:** 1 Department of Geriatric Medicine, Oslo University Hospital, Ullevaal, P.O. Box 4956, 0424 Oslo, Nydalen Norway; 2 Faculty of Health Sciences, Department of Health and Care Sciences, UiT The Arctic University of Norway, P.O. Box 385, 8505 Narvik, Norway; 3 Department of Public Health – Research Unit for Nursing and Healthcare, Department of Science in Nursing, Aarhus University, 8000 Aarhus, Denmark; 4 Faculty of Health Sciences and Social Care, Molde University College, P.O. Box 2110, NO-6402 Molde, Norway; 5 Hammel Neurorehabilitation Centre and University Research Clinic, 8450 Hammel, Denmark; 6 Department of Clinical Medicine, Aarhus University, C, 8000 Aarhus, Denmark; 7 Inland Norway University of Applied Sciences, P.B. 400, 2418 Elverum, Norway; 8 The Faculty of Nursing and Health Sciences, Nord University, P.B. 1490, 8049 Bodø, Norway; 9 University of Oslo, P.O. Box 1130, 0318 Oslo, Blindern Norway; 10 Institute of Health and Society and Research Center for Habilitation and Rehabilitation Services and Models (CHARM), University of Oslo, P.O. Box 1130, 0318 Oslo, Blindern Norway; 11 Oslo Metropolitan University, P.O. Box 4, St. Olavs plass, 0130 Oslo, Norway

**Keywords:** Bias, Complex interventions, Control groups, Process evaluation, Research design, RCT (randomized controlled trials), Rehabilitation research, Stroke, Usual care

## Abstract

**Background:**

A commonly applied control condition in trials evaluating complex interventions in rehabilitation research is “usual care.” The main challenge is to ensure that the control group receives genuine usual care as delivered in everyday clinical practice. The assessment interviews and dialogues with the data collectors may influence the control group participants’ reflections on their condition and adjustments. This represents a threat to the internal validity of the trial. Thus, the aim of this study was to explore the perceived study-induced influence of assessment interviews on the adjustment of the members of a control group in a randomized clinical trial. The aim of the trial was to test a dialogue-based psychosocial intervention aiming at promoting the psychosocial well-being and adjustment of stroke survivors.

**Methods:**

Fifteen participants in the control group of a multicenter stroke rehabilitation trial participated in narrative semi-structured interviews. Ricoeur’s interpretation theory guided the analysis.

**Results:**

The perceived study-induced influence of the assessment interviews on the adjustment process of members of the control group varied considerably. The results demonstrated that the assessment interviews facilitated some participants’ feelings of control and their ability to cope. Other participants’ statements indicate that they relied on their existing personal capacity to cope and adjust and that the assessment interviews did not make any difference either on their coping ability or on their process of adjustment.

Five themes were identified that described the perceived study-induced influence of the assessment interviews in the control group. The themes illustrated that the assessments served as a safety net, enhanced awareness and understanding, encouraged seeking support, allowed the opportunity to vent disappointment, or did not make any difference either way.

**Conclusions:**

RCT assessment interviews may influence the adjustment process and represent a serious problem in measuring interventions over time in trials of complex interventions in rehabilitation research. To uphold rigor and stringency, the usual care control conditions should be thoroughly assessed and described. Informing participants only about the treatment they were allocated to receive might counteract the potential to dilute the difference between the two arms of the trial.

**Trial registration:**

ClinicalTrials.gov NCT02338869. Registered on October 4, 2014

**Supplementary Information:**

The online version contains supplementary material available at 10.1186/s13063-021-05765-w.

## Background

A well-designed (randomized) controlled trial should ensure that a positive result is due to the intervention alone and not to other factors or simply chance [1, 2]. If all participants are treated in exactly the same way, apart from the intervention provided to the intervention group, then the observed differences can be attributed to the intervention [3]. “Usual care” (also known as standard care, treatment-as-usual, or routine care) is a commonly used control condition in randomized controlled trials [2, 4, 5]. However, the descriptions of what constitutes usual care in trials are often unclear and might encompass a variety of practices that are difficult to discern [3, 5, 6] and that may be too variable to clearly characterize the control condition [7].

A gradual shift in usual care practices has been found during several multicenter trials of complex interventions [3, 8, 9]. Changes in usual care have been identified in trials of stroke rehabilitation [8, 9], in intervention trials aimed to prevent falls in nursing homes [3], interventions for young people with type 1 diabetes [5], and in trials of complex interventions in mental health [10]. Factors contributing to changes were communication and networking among clinicians during the trials or the control group participants being treated by multiple clinicians who were also trained in the active intervention [3, 9, 10]. General changes in standard treatment based on newly emerging evidence may also cause changes during the course of a trial [5]. If trial participants in the control group are aware of the content of the intervention, this may alter their behavior and create bias [3, 11]. A main challenge is to ensure that the control group receives genuine usual care as delivered in everyday clinical practice [12].

Considering the complex nature of stroke recovery and adjustment [13–15], the usual care control conditions in stroke rehabilitation trials might also be complex and variable. The control group participants’ reflections on their condition may also change because of the interviews and the questions raised during the trial assessments. These factors may affect their adjustment and potentially affect the internal validity of a randomized controlled trial (RCT). Thus, during the course of a trial, the usual care delivered to control group participants may change and differ from the usual care delivered in everyday practice stipulated in the protocol. This deviation from the protocol might represent a study-induced influence with the potential to dilute the difference between the intervention and the control group and, as such, represent a threat to the internal validity of the trial [3, 8–10].

This study is part of a process evaluation alongside an RCT [16]. The study was conducted to gain an in-depth understanding of the participants’ experiences of being allocated to the control group and of their adjustment process after stroke [16]. Thus, the aim of this study was to explore the perceived study-induced influence of assessment interviews on the adjustment of the members of a control group in an RCT.

## Methods

### Context of the study

The informants in our study had been participants of the control group in a multicenter RCT that tested a dialogue-based psychosocial intervention aimed at promoting the psychosocial well-being and adjustment of stroke survivors. The control group participants received usual care. The participants in the intervention group received an intervention that consisted of eight 1- to 1 ½-h dialogue-based sessions between the stroke survivor and a trained intervention personnel (nurse or occupational therapist) who acted as coaches. To support the dialogue, each session was guided by evidence-based work sheets addressing significant issues and concerns for people with stroke (bodily and emotional challenges, social relationships, activities and existential issues). The sessions were mainly performed in the patients’ homes. The first session took place within 1 month after the stroke and the last within 6 months post-stroke. The intervention personnel were certified following a 3-day training program consisting of lectures, practical training, and group discussions. In addition, the intervention personnel were supervised during the intervention period. Further description of the intervention is outlined in the protocol paper [16].

Our hypotheses were as follows: (1) stroke survivors in the intervention group would experience significantly higher levels of psychosocial well-being and lower levels of depressive symptoms and anxiety (measured by GHQ-28) than stroke survivors in the control group at 6 and 12 months post-stroke and (2) stroke survivors in the intervention group would experience significantly higher levels of sense of coherence (measured by SOC-13) and health-related quality of life (measured by SAQOL-39) than stroke survivors in the control group at 6 and 12 months post-stroke .

The RCT included 322 stroke survivors; 166 were randomized to the intervention group and 156 to the control group. Data were collected from both groups by means of a standardized test battery (Additional file 1) at baseline (T1), 6 months post-stroke (T2), and 12 months post-stroke (T3). However, contrary to our hypothesis, no significant differences between the intervention group and the control group were demonstrated on the outcome measures, either at 6 months or at 12 months post-stroke [17, 18].

Blinded data collectors, nurses and occupational therapists, performed the assessment interviews in the RCT. The data were collected face-to-face in individual, structured assessment interviews, mainly in the participants’ homes. The data collectors underwent training, including a technical component—that is, the use of a web-based questionnaire on a tablet, an electronically available test battery, practical information with a written training package combined with individual training, guidance, and follow-up when needed. The data collectors were instructed to adhere to the questions of the test battery and to administer the questions in the designed, standardized order. They were instructed not to give advice related to diagnosis and treatment and especially not to interfere with the tasks and responsibilities of the community health care personnel. Nevertheless, they were not instructed to refrain from dialogue with the participants about their conditions and their concerns.

### Participants and recruitment

The participants of the actual study were recruited from the control group of the RCT, had sufficient cognitive functioning to provide informed consent and to participate, and understood and spoke Norwegian. Exclusion criteria used in the trial included moderate to severe dementia, serious somatic or psychiatric disease, significant impressive aphasia or severe expressive aphasia [16]. A reiterative purposive sampling procedure was applied [19] based on demographic and stroke-related characteristics. Upon completion of their participation in the RCT, twenty-eight members of the control group were invited by letter with a stamped addressed return envelope to participate in this qualitative part of the process evaluation. Sixteen participants gave their informed consent by returning the consent by post. A lack of response was recorded as a lack of consent. In accordance with research ethics regulations in Norway, those who did not respond were not asked about their reasons for refraining from participation One of the participants who consented was subsequently excluded because his health condition deteriorated. Six women and nine men aged 29—88 years, participated in this study. Characteristics of the participants are shown in Table 1.

**Table 1:** Characteristics of the participants

Baseline demographics (***n*** = 15)	***N*** (%)
Age (mean)	63
Sex	
Female	6 (40)
Male	9 (60)
Living conditions	
Living with someone	12 (80)
Living alone	3 (20)
Caring for children	
Yes	4 (26)
No	11 (74)
Education	
Compulsory schooling	2 (13)
Upper secondary school	9 (60)
College university	4 (27)
Work life pre stroke	
Disability pension	3 (20)
Retired	5 (33)
Retired working part time 50–60%	2 (13)
On job search	1 (7)
100% employed	4 (27)
Work life one year post stroke	
Disability pension	4 (27)
Retired	8 (53)
On sick leave	2 (13)
100% employed	1 (7)
Rehab services at 12 months post stroke	
None	11 (73)
Physiotherapy, occupational therapy	1 (7)
Physiotherapy	2 (13)
Physiotherapy, speech therapy, home care nursing	1 (7)
Stroke etiology, location	
Infarct/bilateral	1 (7)
Infarct right side	5 (33)
Infarct left side	7 (46)
Infarct side unknown	1 (7)
Hemorrhage left side	1 (7)
NIHSS score	
Mild (0–5)	5 (33)
Moderate (6–10)	4 (27)
Severe ≥ 11 moderate to severe stroke	1 (7)
Unknown	5 (33)
Speech impediment	
Yes	6 (40)
No	8 (53)
Unknown	1 (7)

### Interviews

All authors took part in developing the interview guide and cooperated closely in planning of the interviews. Nine of the authors conducted the interviews from July 2016 to June 2017. The interviews were primarily narrative in style to encourage participants to convey their illness experiences and experiences of the assessment interviews in the RCT [20]; see the interview-guide (Additional file 2). Interviews were conducted in a setting chosen by the participant and lasted from 17 to 76 min (median 43 min). The interviews were tape-recorded and transcribed verbatim by professional transcribers. The transcribers who listened repeatedly to the tape-recordings to secure the accuracy of the transcripts were external to the research team.

### Analysis

Ricoeur’s interpretation theory [21, 22] guided the analysis in three steps: naïve interpretation, structural analysis, and critical interpretation. According to Ricoeur, the naïve reading is the immediate interpretation of the material [21]. Five members of the research team (i.e., the working group) read all the transcripts and gained an overall understanding of the data material. Through independently reading and re-reading all the interview transcripts several times, separately and as a whole, an overall interpretation of the possible study-induced influence of the assessment interviews was made by the working group members (MM, GK, ASE, SA, and LA). Next, the interview texts were distributed among the working group members, who then performed the structural analysis. Sentence by sentence, text sequences were interpreted in the context of the text as a whole, what was said in the text, and what the text talked about [21]. This part of the analysis worked through an *explanation* “from what it says, to what the text talks about” [21]. In relation to what the text possibly talked about, we sought contributions from all members of the research team regarding what sentences could mean. According to Ricoeur [21], the third step of the analysis is the development from the explanation in the structural analysis to a comprehensive understanding of the whole. With the naïve interpretation and the results of the structural analysis in mind, the whole research team was involved in achieving the most probable interpretation.

### Ethics

The Regional Committee for Medical and Health Research Ethics (Case number: 2013/2047) and the privacy protection ombudsman (Case number: 2014/1026) responsible for the hospitals involved in the RCT approved the study (Trial Registration: ClinicalTrials.gov (https://clinicaltrials.gov/) with trial number NCT02338869. Oral and written informed consent, also adjusted for patients with aphasia [23], was collected from all participants in the study. Prior to the interviews, information about the study and the participants’ rights was repeated. All research procedures complied with the Declaration of Helsinki [24].

## Results

Participants’ descriptions of their illness experiences and of their adjustment process varied considerably. Most participants had minor to moderate impairments, and one had a moderate to severe stroke, based on the National Institutes of Health Stroke Scale (NIHSS) score of stroke severity [25] (Table 1). The NIHSS scores of five of the participants were unknown.

The results illustrated the participants’ drive and struggle to recover and regain their perception of their pre-stroke self. Participants’ efforts to cope and adjust were illustrated in their descriptions of the assessment interviews. They took advantage of the opportunities to gain information and support during the dialogues with the data collectors. Thus, their efforts to adjust and cope were extended into the assessment interviews.

Several statements illustrated that the assessment interviews in various ways influenced participants’ reflections on their condition and that participation had fulfilled some of their unmet needs. The dialogues with the data collectors enhanced the participants’ awareness and understanding of their post-stroke condition and gave them the opportunity to learn something new. The perception that the assessment interviews did not make any difference either on their coping ability or on their process of adjustment was expressed by participants who had experienced mild strokes and spontaneous recovery. These participants emphasized setting their own goals, support from family and friends, and a desire to support research to the benefit of other stroke survivors.

In the structural analysis, we identified five themes describing control group participants’ perceptions of the study-induced influence of the assessment interviews on adjustment following stroke. The themes illustrated that the assessments: served as a safety net, enhanced awareness and understanding, encouraged seeking support, allowed the opportunity to vent disappointment, or did not make any difference either way. The themes are further described in the following section.

### The assessments served as a safety net

Some statements illustrated that participation was perceived as a safety net in the case of a new stroke. By being included in the study, it seemed easier to manage feelings of worry and loneliness. One participant described feeling relief at being recruited to the trial and was convinced that the data collectors would notify the health care services if a crisis arose. Since someone outside his family understood how he felt, he did not feel entirely alone. The importance of access to professional advice was emphasized:

“It’s important to be reminded… that you have an opportunity to talk to someone about what’s happened. And that you realize that it’s something you should take into consideration and be aware of. And maybe alert someone if you’re uneasy. So you’re not reluctant to notify someone who knows and tell them about your worries. It’s easy to sweep problems under the carpet.”

Other statements illustrated that participation implied an advantage, extra attention, and protection in case something unexpected happened. One participant expressed that she expected general protection that extended beyond the context of the data collectors and the assessment interviews.

“I really think it’s been okay to participate. If something happened.. then I had … contacts… then someone would be notified via this project… maybe.”

Feelings of safety and protection seemed to facilitate the participants’ ability to cope, which, in turn, might have influenced their adjustment process.

### The assessments enhanced awareness and understanding

The opportunity to talk with a friendly, interested, and capable person about their illness experiences was important. The assessment interviews met the participants’ needs for information and enhanced reflection, awareness and understanding. Some statements illustrated participants’ anxiety and fear of another stroke and showed that this fear had not been sufficiently addressed in the hospital or in consultations with their regular doctor:

“I didn’t talk to the doctor about it. But I got to know… that it can happen again”…. “But the fact is, I’d expected him to show me a little more personal interest.”

Experiencing the stroke made this participant more introverted, and he appreciated the opportunity to reflect on his condition with a professional:

“I really appreciated the opportunity to discuss [stroke-related issues]. Otherwise, I’d have been sitting alone without the chance to reflect during this convalescence period. So, I think the meetings with the data collector encouraged me to think… and I think I’ve managed this process better than I would have done if I hadn’t taken part in the trial.”

It is notable that the assessment interviews and dialogues were perceived as *“discussions*,” and thus as an opportunity to converse about important questions:

“The meetings helped me to understand much more why things work and don’t work.”

Other statements illustrated that the interview sessions were perceived by the participants as an opportunity to receive emotional support and to have meaningful dialogues about their condition:

“I think it’s really been a pleasure that they visit you at home, ask you about your experiences and how you feel. I think that’s really nice. Knowing that there is some kind of follow-up.”

The questions in the test battery helped this participant recognize her own post-stroke situation:

“You become aware of things you otherwise wouldn’t reflect on. So in a way that’s helped me [laughter] … your self-concept, you know. You reflect on what’s happened. You can reflect more honestly about your condition.”

Another participant who had lived with a chronic illness before the stroke incident stated that he generally really hated to talk about illness. He experienced the dialogues with the data collector as something completely different:

“I hate talking about illness, but this is something completely different. It has to do with understanding it and becoming more conscious of it so that you can try not to worry too much and talk about it. You get a more sensible approach to [the stroke].”

One participant explained that the assessment interviews helped him become aware of his progress between the sessions:

“The three interview sessions helped me become more aware of my own progress. I’ve made progress since my previous interview session.”

These statements seem to illustrate that the assessment interviews opened up dialogues and reflections different from what was expected from ordinary encounters with health professionals in the community.

### The assessments encouraged seeking support

Several statements illustrated participants’ surprise at some of the questions in the test battery, especially their thoughts regarding whether life after a stroke was worth living. A typical comment among the participants was the assumption that “*other people*” might react negatively to such questions:

“Obviously, I know that many people react when there’s talk about death …Because you don’t feel you’re worth anything anymore, or you feel weaker than you thought you were … you make a decision that you don’t want to be part of it any longer.”

At the same time, these participants tended to maintain that they did not perceive that kind of question as negative.

“But I didn’t feel uncomfortable answering the questions. It’s possibly because you’ve experienced so much. Seen some terrible things; life is fragile.”

One participant perceived such questions as irrelevant and “*stupid*,” and another stated that some questions were intrusive and possibly “*dangerous*.” He expressed concern that, in general, such kinds of questions could trigger suicidal thoughts among other vulnerable participants, and he asked if the data collectors could involve advisors in case some of the participants had suicidal thoughts. However, this participant described how the questions about his mental state had encouraged him to consult a psychologist:

“But [these questions] started thought processes. That was when I started to think that I ought to talk to a psychologist. Because there were some thoughts that weren’t positive.”

It therefore seemed that these questions in the test battery had encouraged him to seek support for a situation he had not previously been aware of.

### The assessments allowed the opportunity to vent disappointment

Several statements illustrated a sense of disappointment associated with being randomized to the control group. One participant expressed envy of other participants who had been lucky enough to be in the intervention group.

“I feel a bit jealous, but some people got to be in the control group as well.”

Other statements illustrated a sense of disappointment about certain aspects of their participation in the control group. One participant was disappointed because he had expected information from the data collectors about his diagnosis and prognosis:

“There were some things I was wondering about. I was asking about several things, about my disease, and about how long it would take to get well and get back to work.”

Participation in the trial seemed to trigger expectations to fulfill unmet information needs. Thus, awareness of the existence of the intervention arm might have triggered reflections on their condition, including awareness of unmet information needs.

### The assessments did not make any difference either way

Several statements expressed by participants who had experienced mild strokes and felt that they had recovered spontaneously suggested that it was “okay” to participate and that they subsequently had not at all reflected upon the interview sessions. One participant said:

“Follow up is okay, but I’ve felt well all the time, so I cannot say I’ve had any personal benefit.”

Other statements emphasized feelings of luck and relief at having survived the stroke without major impairments.

“I’ve been lucky, I recovered quickly. I just answered the questions, nothing more, and I haven’t thought about it afterwards.”

Talking about the recovery and answering the questions in the test battery was not perceived as a burden, and these statements illustrated that some participants did not find the questions inappropriate or intrusive.

“It was perfectly fine. None of the questions were unpleasant.”

Other statements highlighted support from family and friends or their internal coping strategies as their capacity for problem solving and maintaining a proactive approach. One explained the latter point:

“To me, this study did not make a difference either way. I’ve drawn up subgoals and milestones all along and made a training program. I try to practice things that I know I have problems.”

Participants who indicated that they did not experience any personal benefit also emphasized the importance of contributing to research for the benefit of other persons with stroke.

“Not for me, but I think it might be beneficial for research. They see, OK she’s done well, she’s in that category. And then you have others who are not doing well.”

The assessment interviews did not seem to reveal any reflections on this participant’s condition or any new thoughts about the implications for her life post-stroke.

## Discussion

The results of this process evaluation illustrate the challenges associated with ensuring that control group participants do not receive additional support beyond the usual care delivered in clinical practice when participating in an RCT. The experiences that our control group participants reported in relation to the assessment interviews correspond closely to the intentions of the psychosocial intervention tested in this RCT, namely, to promote awareness, reflections, and feelings of support. Furthermore, the participants’ statements illustrated that therapeutic content was perceived to be a substantial part of the assessment interviews for many of the participants. Other participants, however, stated that the assessment interviews did not influence their reflections on their condition or their adjustment process.

When participants described their experiences of inadequate follow-up and shortcomings in hospital and community health care, they also emphasized the influence of the assessment interviews. Thus, participants’ need for support might be related to their personal ability to cope and adjust, to the quality of their family network, and to possible gaps in the quality of health care services. The assessment interviews encouraged some participants to seek professional support. This can be considered a utilization and an extension of the usual care received outside the setting of the study. This illustrates that usual care might be highly variable, including “non-study care,” such as private care sought on the participants’ own initiative [2, 26]. The results illustrate the disappointment of not being allocated to the intervention group. This perception also facilitated awareness of unmet information needs that might have encouraged participants to seek support outside the scope of the trial. As illustrated in this study, the rigor of an RCT in rehabilitation research may be challenged by the possible study-induced influence of individual assessment interviews. It is challenging to differentiate between adjustment caused by natural recovery and participants’ own capacity to cope and adjust compared to influences on adjustment caused by the assessment interviews and dialogues with the data collectors.

Ideally, in experiments conducted in stringent and tightly controlled conditions, experimental manipulation would be the only difference between groups formed by random allocation [2]. However, in complex intervention studies performed in participants’ natural environments, the opportunity to design control conditions is limited, as this is dependent on the delivery of usual care in the particular community. In the current study, the presumption of the design was that responding to the test battery would have a limited influence on the participants’ adjustment process in terms of delivering elements of the active ingredient to the controls as delivered to participants in the intervention arm. The test procedure implied that the data collectors were instructed to adhere strictly to the test battery. However, they were not instructed not to respond to any questions from the participants. Participants’ unmet needs for information and support were demonstrated in this study. This is consistent with the intervention group that also stated that the dialogues with the intervention personnel facilitated reflection and filled the gap of unmet needs [27]. Further, the questions in the test battery might have been perceived as an invitation to reflect on their condition and existential issues. Such factors could have blurred the differences between the intervention group and the control group. Thus, the assessment test procedure might threaten the internal validity of the RCT [28] and possibly influence the results of the trial.

One striking result of this study was that assessment interviews of 30–45 min with a dedicated professional on three occasions seemed to facilitate these participants’ reflections on their condition. Our results suggest that mere attention, listening, and dialogue with a professional might have had an impact on the adjustment process of the controls. Nonspecific factors, such as “human interaction variables,” clinicians’ warmth, and empathy, may have a substantial impact on the outcome of an RCT, as previously shown [28]. It might be challenging to balance the participants’ need for information and support with the methodological and research ethical guidelines guiding RCTs, that is, to avoid influencing the participants’ reflections on their condition and their adjustment process. To protect the control group participants from the influence of face-to-face assessment interviews, either telephone interviews or answering the questionnaires on their own would have been alternatives. However, one should consider the obvious challenges associated with assessments of stroke survivors with varying degrees of impairment. In that case, the planning and implementation of the assessment interviews should have been subject to meticulous attention and preparation.

We have shown that the assessments had the potential to influence adjustment and thus dilute the differences between the two groups. However, the participants in both groups received one or more rehabilitation services adjusted to their individual needs, and the proportion was high both at baseline and at 12 months [17]. Both trial arms were also assessed at T1, T2, and T3. Considering that the participants in the study arm received both intervention and assessments, there might be an unknown compound effect of rehabilitation services, intervention, and assessments. Thus, whether the assessment interviews introduced a dilution of the treatment contrast in this trial remains questionable.

Some authors propose that disclosure of information about the trial should be restricted and that the participants should be given neutral information [29]. If the controls were unaware of the existence of the intervention arm, this might have diminished the risk of study-induced behavior change [12]. The Zelen design, which involves obtaining consent from participants after randomization, has been suggested to minimize these kind of threats in RCTs [30]. Only those who had been randomized to the experimental group would then be asked to consent to participate in the trial, while the controls would remain uninformed [30–35]. However, to withhold such vital information would be considered ethically unacceptable from a research ethics perspective [12, 36, 37]. In a modified two-stage consent design, those assigned to the control group would receive the usual care, and they would know that other people received different care but without knowing what that care entailed [38].

Applying a Zelen consent design modification to our study, the control group participants would neither be informed nor aware of the existence of the intervention. But they would be informed about, and could then consent to participate in, the assessment interviews at the prescribed three points in time, T1, T2, and T3. This approach might be perceived as meaningful by the participants, both as a potential confirmation of their own progression and adjustment and as an opportunity to contribute to research for the benefit of other persons with stroke. Simultaneously, one would avoid the disappointment of not being allocated to the intervention group. This might be considered an ethically sound approach, especially given that this trial does not imply any risk for the participants, either for those in the intervention group or for the controls. However, out of respect for research participants’ autonomy, obtaining informed consent has been considered a cornerstone of research ethics [37, 39]. At the same time, taking into account research participants’ vulnerability, the importance of building and maintaining long-term trusting relationships between researchers and participants has been highlighted [40].

### Methodological considerations

Our study has limitations. The randomization in this trial should ensure that any positive result of the intervention was due to the treatment and not on other factors. Our sample was selected to include participants with various socio-demographic and stroke-related characteristics. However, in view of the limited sample size and the qualitative nature of this study, we cannot conclude that the results were representative of all the control group participants in the RCT. Participants’ disclosure of their experiences of illness and of participating in the RCT revealed rich and nuanced data. This is considered a strength of the study. Considering some of the participants’ stroke-related impairments, recall bias might have influenced their perceptions and judgments of the influence of the assessments. The interviews were performed in retrospect after the completion of the T3 assessment interviews. The participants might have had difficulty recalling the assessment interviews and the dialogues with the data collectors, which could have influenced the results.

All the researchers were involved in the development of the interview guide and in the analysis process. The multicenter trial recruited participants from eleven hospitals located in different geographical locations in Norway. Thus, the participants in this study were recruited from different parts of the country which entailed long distances. Of practical reasons as many as nine researchers conducted the qualitative interviews. Six of the interviewers had participated as intervention providers but did not interview any of the participants they had visited and followed up in that capacity. Several other interviewers had participated in the development of the trial and had worked as project coordinators. Some interviewers had acted as data collectors in the RCT by means of the standardized test battery, although they had not previously collected any data from the participants in this study. Some of the interviewers’ extensive knowledge of and involvement in the trial might have strengthened the depth and nuances of the interviews. To avoid the preconception that the interviewers should influence the interviews, analysis, etc., we composed a broad team with various relationships with the intervention.

## Conclusion

The results of this process evaluation demonstrated how the assessment interviews had the potential to facilitate participants’ adjustment after stroke and encourage them to utilize usual care. Elements that corresponded closely with the active ingredients of the intervention were identified by some of the participants in the control group. Training and supervision of data collectors and alternative approaches to assessment are important factors to consider reducing threats to internal validity. The usual care control conditions should be thoroughly assessed and described. Informing participants only about the treatment they were allocated to receive might counteract the potential to dilute the difference between the two arms of the trial.

## Supplementary Information

**Additional file 1:** Measurements.

**Additional file 2:** Interview guide

## Data Availability

The datasets generated and analyzed during the current study are not publicly available due to strict ethical regulations in Norway but may be available from the corresponding author on reasonable request.

## Abbreviations

RCT: Randomized controlled trial
GHQ-28: General Health Questionnaire – 28
SOC-13: Sense of Coherence –13
SAQOL-39: Stroke and Aphasia Quality of Life Scale – 39
NIHSS score: National Institutes of Health Stroke Scale score
