# Supplementary material for: Perceived study-induced influence on the control group in a randomized controlled trial evaluating a complex intervention to promote psychosocial well-being after stroke: a process evaluation
Source: Trials. 2021 Nov 27;22:850. doi: 10.1186/s13063-021-05765-w (PMC8627040; doi:10.1186/s13063-021-05765-w)
Supplement: Supplementary file 1 — Additional file 1: Measurements. [file 13063_2021_5765_MOESM1_ESM.docx]

**Measurements**

**Primary outcome of the RCT:**

**The General Health Questionnaire (GHQ-28)** is a 28-item questionnaire measuring psychosocial wellbeing.

**Primary outcome:** Emotional distress

**Description:** Scaled 28-item self-report questionnaire measuring emotional distress. Four subscales identified in psychometric tests (somatic symptoms, anxiety and insomnia, social dysfunction and severe depression.

**Assessment:** T1, T2, T3

**Secondary outcomes of the RCT:**

**The Stroke Aphasia Quality of Life Scale (SAQOL-39)** addresses general dimensions of health-related quality of life.

1. **Health-related quality of life**

**Measure:** Stroke and Aphasia Quality of Life Scale-39 generic stroke version (SAQOL-39g) 38, 39

**Description:** Disease-specific health-related quality of life scale, measures patient’s perspective of stroke’s impact on ‘physical’, ‘psychosocial’ and ‘communication’ domains.

**Assessment:** T1, T2, T3

**The Sense of Coherence Scale (SOC-13)** measures psychosocial well-being.

1. **Sense of coherence**

**Description:** Sense of Coherence scale (SOC-13) Self-report questionnaire, 13 components, measuring the main concepts in the SOC theory; coherence, meaningfulness and manageability. 13 items scored on a Likert scale, ranging from 1–5. Higher scores indicate a stronger SOC.

**Assessment:** T1, T2, T3
