# Supplementary material for: Perceived study-induced influence on the control group in a randomized controlled trial evaluating a complex intervention to promote psychosocial well-being after stroke: a process evaluation
Source: Trials. 2021 Nov 27;22:850. doi: 10.1186/s13063-021-05765-w (PMC8627040; doi:10.1186/s13063-021-05765-w)
Supplement: Supplementary file 2 — Additional file 2: Interview guide [file 13063_2021_5765_MOESM2_ESM.docx]

**Interview guide: To explore the influence of the assessment interviews on control group participants in a complex psychosocial intervention study after stroke**

**Introduction**

- To inform about the purpose of the interview
- To make clear that there are no wrong or right answers
- To emphasize the importance of sharing their experiences
- Obtain informed consent

**Theme 1**

**Participants’ experiences from illness onset until interview and their experiences regarding the assessment interviews**

**First question**

**Can you please tell me about your experiences from the onset of your illness until today?** The interviewer should not interrupt the narrative. If necessary, ask follow-up questions:

- Changes in everyday life
- Changes in emotions
- Social life – being with others
- Work and leisure activities
- Body and health
- Self-image
- Emotional life
- To meet challenges after stroke

**Second question**

**Can you please tell me about your experiences of taking part in the project?**

The interviewer should not interrupt the narrative. If necessary, ask follow-up questions:

- To talk about one’s situation
- Changes in the understanding of the illness during the project
- Thoughts about the needs of the relatives and their possible role in the project
- Having the opportunity to talk about one’s own situation
- Positive consequences of participating in the project
- Negative consequences of participating in the project

**Theme 2**

**Experiences with the arrangement of the project**

**First question: Can you please tell me about your experiences of the assessment interviews?**

If necessary, ask follow-up questions:

- How did you experience answering the questions three times?
- Were there too many questions / appropriate questions?
- How did you experience the questions that were asked?
- Was the length of each meeting suitable / too short / too long?

**Question 2:** How did you experience the collaboration between you and the interviewer?

**Question 3:** How did you experience participating in three interviews without being offered extra follow-up?

**Theme 3**

**Other comments**

**First question:** Do you think there is a need for follow-up beyond what you received, and how do you think such an offer should be?

**Second question:** is there anything you want to add that we haven’t talked about?
